# Rapid Microscopy and Use of Vital Dyes: Potential to Determine Viability of *Cryptococcus neoformans* in the Clinical Laboratory

**DOI:** 10.1371/journal.pone.0117186

**Published:** 2015-01-27

**Authors:** Brendan J. McMullan, Desmarini Desmarini, Julianne T. Djordjevic, Sharon C-A. Chen, Michael Roper, Tania C. Sorrell

**Affiliations:** 1 Centre for Infectious Diseases and Microbiology, Westmead Millennium Institute, University of Sydney at Westmead Hospital, Westmead, NSW, Australia; 2 Centre for Infectious Diseases and Microbiology Laboratory Services, Westmead Hospital, Westmead, NSW, Australia; 3 Marie Bashir Institute for Infectious Diseases and Biosecurity, University of Sydney, Westmead, NSW, Australia; 4 ICPMR–Pathology West, Westmead Hospital, Westmead, NSW, Australia; 5 Discipline of Infectious Diseases and Immunology, Westmead Clinical School, Sydney Medical School, The University of Sydney, Westmead, NSW, Australia; University of Minnesota, UNITED STATES

## Abstract

**Background:**

*Cryptococcus neoformans* is the commonest cause of fungal meningitis, with a substantial mortality despite appropriate therapy. Quantitative culture of cryptococci in cerebrospinal fluid (CSF) during antifungal therapy is of prognostic value and has therapeutic implications, but is slow and not practicable in many resource-poor countries.

**Methods:**

We piloted two rapid techniques for quantifying viable cryptococci using mixtures of live and heat-killed cryptococci cultured *in vitro*: (i) quantitative microscopy with exclusion staining using trypan blue dye, and (ii) flow cytometry, using the fluorescent dye 2′-7′-Bis-(2-carboxyethyl)-5-(6)-carboxyfluorescein, acetoxymethyl ester (BCECF-AM). Results were compared with standard quantitative cryptococcal cultures. Quantitative microscopy was also performed on cerebrospinal fluid (CSF) samples.

**Results:**

Both microscopy and flow cytometry distinguished between viable and non-viable cryptococci. Cell counting (on log scale) by microscopy and by quantitative culture were significantly linearly associated (p<0.0001) and Bland-Altman analysis showed a high level of agreement. Proportions of viable cells (on logit scale), as detected by flow cytometry were significantly linearly associated with proportions detected by microscopy (p<0.0001) and Bland-Altman analysis showed a high level of agreement.

**Conclusions:**

Direct microscopic examination of trypan blue-stained cryptococci and flow-cytometric assessment of BCECF-AM-stained cryptococci were in good agreement with quantitative cultures. These are promising strategies for rapid determination of the viability of cryptococci, and should be investigated in clinical practice.

## Introduction

Cryptococcal meningitis (CM) is a life-threatening infection, for which the identification of rapid practical biomarkers of prognosis and response to therapy is problematic. In therapeutic trials in patients with HIV-associated CM, cerebrospinal fluid (CSF) culture negativity within 14 days and the rate of fall in viable cryptococci in CSF, expressed as the Early Fungicidal Activity (EFA) have been correlated with clinical outcomes [1–5]. However, quantitative cultures are relatively slow and may not be practicable, especially in resource-limited settings, where the vast majority of cases occur. Serial measurement of cryptococcal antigen titres correlates poorly with outcomes[1,6,7]. India Ink staining in HIV-associated CM has been correlated with quantitative CSF cultures [1,2], but can remain positive long after sterilisation of the CSF and viable and dead cryptococci are not distinguished [2,8–10]. Nucleic acid-based amplification methods are sensitive and specific, but are expensive and resource intensive, do not distinguish live and dead cryptococci, and turnaround times are suboptimal.

CSF microscopy is rapid, simple, quantitative and widely available. Dyes for vital staining of cryptococci have not been formally evaluated in a clinical setting [8–11], but exclusion of trypan blue is used to identify viable cells of the yeast, *Saccharomyces cerevisiae*, and has been used for *Cryptococcus gattii* cultured *in vitro* [11,12]. Cryptococcal viability has also been assessed by flow cytometry using the dye 2′-7′-Bis-(2-carboxyethyl)-5-(6)-carboxyfluorescein, acetoxymethyl ester (BCECF-AM), which is taken up by cells and converted to its fluorescent form by the action of cellular esterases [12,13]. This dye is a widely used indicator of intracellular pH that has been used to assess viability in mammalian cells [13–15]. Furthermore, flow cytometry is widely used in clinical haematology, immunology and research, but has had limited use in the clinical microbiology laboratory to date.

We hypothesised that quantification of viable cryptococci by the use of vital dyes might be a practicable, cheap and rapid alternative to quantitative culture in clinical laboratories. We also hypothesised that quantification of viable cryptococci by flow cytometry constitutes a rapid alternative to quantitative culture in clinical laboratories within developed settings.

## Materials and Methods

### Cryptococcal sample preparation

*C*. *neoformans* var. *grubii* strain H99 (obtained from W. Meyer, Molecular Mycology Laboratory, Westmead Hospital) was cultured on Sabouraud’s Dextrose Agar (SDA) for 48 hours, harvested and adjusted to a concentration of 1 McFarland by nephelometry. This corresponded to between 1.5 ×10^6^ CFU/mL and 4.7 ×10^6^ CFU/mL for individual samples (as confirmed by quantitative cultures). This concentration is similar to that in CSF samples from HIV-infected patients with CM [14–17]. Heat-killed cryptococci were prepared by incubation at 56–60°C for 60 min as confirmed by absence of growth on SDA agar and universal uptake of trypan blue. Different mixture ratios of 100:0, 50:50, 10:90, 1:99, 0:100 live to killed cryptococci were prepared (at dilution 1 McFarland). We originally intended to use heat-killed cells only but included non-viable cells killed by nutrient starvation later when we observed unexpectedly bright fluorescence in heat-killed cryptococci, to examine whether this appearance was an artefact of heat killing. Cultures killed by nutrient starvation were incubated for 5 days or 14 days on SDA alone at 30 degrees; lack of viability confirmed by the absence of growth on SDA and uptake of trypan blue.

### Clinical samples

Two additional clinical samples were used in this study: (1) a CSF sample from a patient with aseptic meningitis was spiked with cryptococci and microscopy counts using trypan blue were compared with quantitative cultures, and (2) a pre-treatment sample from a patient with cryptococcal meningitis. Cryptococci were counted and assessed by trypan blue microscopy but there was insufficient CSF left to perform quantitative cultures on this sample.

### Trypan Blue Microscopy by Haemocytometer

For direct microscopy, ratios of 100:0, 50:50, 10:90, 1:99, 0:100 live to killed cryptococci were stained with 0.4% trypan blue (Sigma-Aldrich, Australia) for a minimum of 5 minutes and maximum of 15 minutes. Cells were visualised and counted in a haemocytometer by standard methods. For determination of the percentages of live and dead cryptococci, 200 cells were counted and the number per mL calculated. For comparison with quantitative cultures these counts were expressed as units CFU/mL to facilitate statistical analysis (see Statistical analysis).

### Flow cytometry with BCECF-AM staining

For flow cytometry, BCECF-AM, 20 microL, was incubated with 980 microL of live:dead cryptococcal mixtures for 15 minutes. Ratios of 100:0, 50:50, 10:90, 1:99, 0:100 live to killed cryptococci were prepared for analysis as described above. Some samples were tested after washing to remove extracellular BCECF-AM. A minimum of 50,000 events were analysed using the FITC-A channel of a FACS CANTO II Flow Cytometer using FACSDiva software.

In two subsequent experiments, absolute counts of cryptococci were quantified by flow cytometry using TruCount tubes containing fluorescent beads, as per the manufacturer’s instructions (BD Biosciences, California USA)[16].

### Statistical analysis

Microsoft Excel 2011 (Microsoft Corporation, USA) and R version 3.1.1 [18] were used for statistical analyses described in this study. Bland-Altman analysis was used to assess the agreement between measurement methods[19]. The MethComp package in R was used for the Bland-Altman analysis[20]. The analysis of both measurement methods used a model of linked replicates[21]. For comparison of the cell counting methods all results were transformed to log CFU/ml for analysis. Only measurements above 10^4^ CFU/mL, considered as the detection limit of the assay [22,23], were included in the analysis. Cytometry measurements were only available for analysis as percentages and were analysed on logit scale. Prior to the logit transform, measurements of 100% were replaced with (100-ε)% and measurements of 0% were replaced with ε % where ε was the minimum recorded viable cell percentage[24].

## Results

### Trypan Blue microscopy

**Figure 1:**
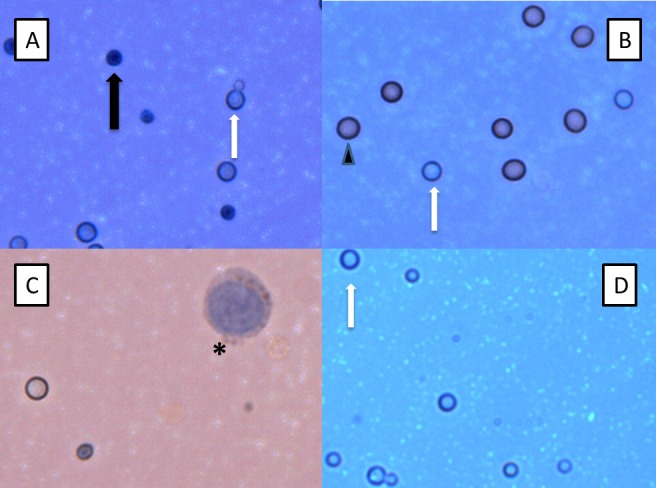
Trypan Blue Microscopy of Cryptococci. Light microscopic examination of Trypan blue-stained cryptococci at 40x magnification readily distinguishes viable (transparent, white arrow) and dead (dark blue, black arrow) cryptococci in *in vitro* (A) and CSF (B-D) samples. Note the presence of budding viable cryptococci in (A). Erythrocytes (arrowhead) are also observed in the CSF sample in (B) alongside viable cryptococci. (B), (C) and (D) are CSF samples obtained before and after dilution with water, respectively, which lyses mammalian cells. The large nucleated cell (asterisk) observed before dilution in (C) is a leukocyte. Many viable cryptococci remain in the CSF after lysis of mammalian cells in (D) (white arrow). The different intensity of background colour is due to adjustment of microscopy lighting, focus and contrast. No colour adjustment has been performed post-photography.

**Figure 2:**
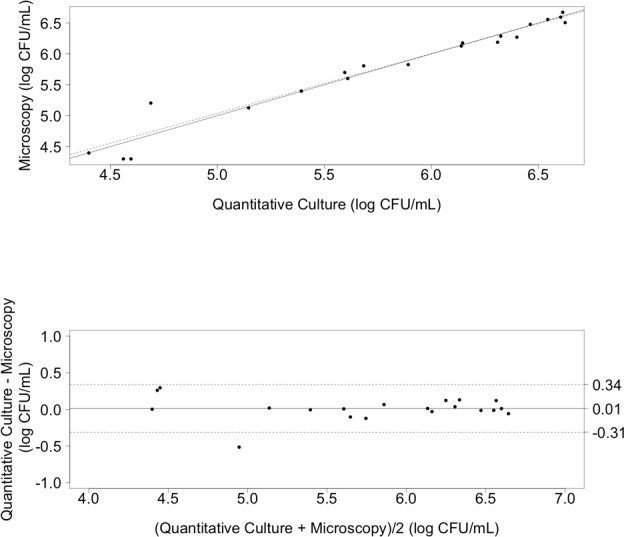
Cryptococcal Counts via Microscopy and Quantitative Cultures. From prepared samples of 100:0, 50:50, 10:90, 1:99, 0:100 live:dead cryptococci. **2A (top). *Scatter plot for Quantitative Culture and Microscopy cell counting methods***. Solid line indicates the reference line. Dotted line indicates the regression line. Counts are for viable cryptococci. **2B (bottom). *Bland-Altman difference plot for Quantitative Culture and Microscopy cell count methods***. The bias (mean difference between measurement methods) is shown as a solid horizontal line and the 95% limits of agreement are shown with dashed lines at +/− 1.96 standard deviations.

Quantification in a haemocytometer coupled with trypan blue staining distinguished viable from non-viable cryptococci in experimental samples, and results were then compared to those obtained by quantitative culture (Fig. 1A). For the comparison of cell counting techniques, data was available for n = 6 samples (separate experiments). Mean log-transformed microscopy counts obtained for the viable mixtures (100:0 live:dead) were 6.47 CFU/mL (95% confidence interval 6.27–6.68) and those obtained for the mean log-transformed quantitative culture counts were 6.49 CFU/mL (95% confidence interval 6.29–6.69). Between 1 and 5 replicates were available per sample of different mixture ratios of 100:0, 50:50, 10:90, 1:99, 0:100 live to killed cryptococci; the complete data set is detailed in the Table 1 in S1 File. Cell counting by microscopy and by quantitative culture were significantly linearly associated (Fig. 2A, R^2^ = 0.96, p<0.0001). The Bland-Altman analysis (Fig. 2B) showed a high level of agreement between the two measurement methods with a bias of only 0.01 log CFU/mL (SD 0.16). The 95% limits of agreement were-0.31 log CFU/mL and 0.34 log CFU/mL. Within this dataset there was a single outlier.

Quantification by trypan blue microscopy was then applied to two clinical samples. We spiked a CSF sample from a patient with aseptic meningitis with cryptococci. Counts obtained by microscopy were 1.3×10^5^ CFU/mL and by quantitative culture, 2.0×10^5^ CFU/mL (absolute difference 0.19 log CFU/mL). Finally, a pre-treatment CSF sample from a patient who tested positive for cryptococcal meningitis using standard cryptococcal culture and India ink staining, yielded a cryptococcal count of 6.5×10^4^ CFU/mL (76% viable cells) by trypan blue microscopy. However, there was insufficient sample for quantitative culture. Erythrocytes and leukocytes were also present in CSF samples and were readily distinguished from cryptococci using microscopy (Fig. 1B and 2C). However, to ensure that cryptococci are distinguished from lymphocytes and erythrocytes, we demonstrated that both human cell types could be lysed by incubation of the sample with distilled water (1:4 vol/vol), without effecting the integrity or viability of cryptococci (Fig. 1D).

### Flow Cytometry

**Figure 3:**
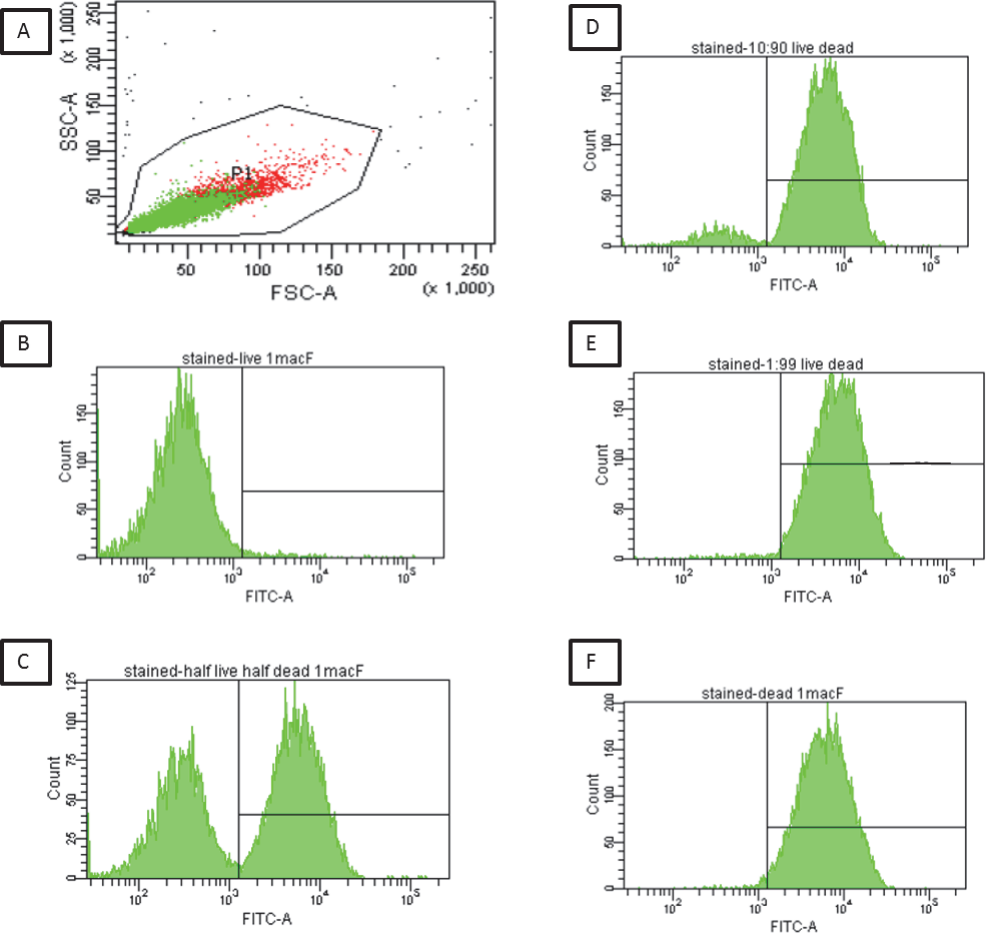
Flow Cytometric Assessment of Cryptococcal Viability using BCECF. (3A-F) Heat-killed and viable Cryptococci were mixed to yield the following ratios of live:dead cells (100:0, 50:50, 10:90, 1:99, 0:100) at a dilution of 1 McFarland and stained with BCECF: A representative scattergram for the 50:50 mixture a dilution of 1 McFarland is shown in (3A), with the gate containing both live and dead cells. Green dots are single cells, which were used for analysis of percentage viability, and red dots are potential doublets or clumped cells. All mixtures yielded a similar forward and side scatter plot, indicating that there was no differentiation between live and dead cells. 3B-E: Histogram plots obtained for mixtures of 100:0, 50:50, 10:90, 1:99, and 0:100 live:dead cells. Viable cells (low fluorescence) are indicated in the left panel and non-viable cells (bright fluorescence) in the right panel. FSC: Forward Scatter; SSC: Side Scatter; FITC-A: Fluorescence in the fluorescein isothiocyanate channel.

**Figure 4:**
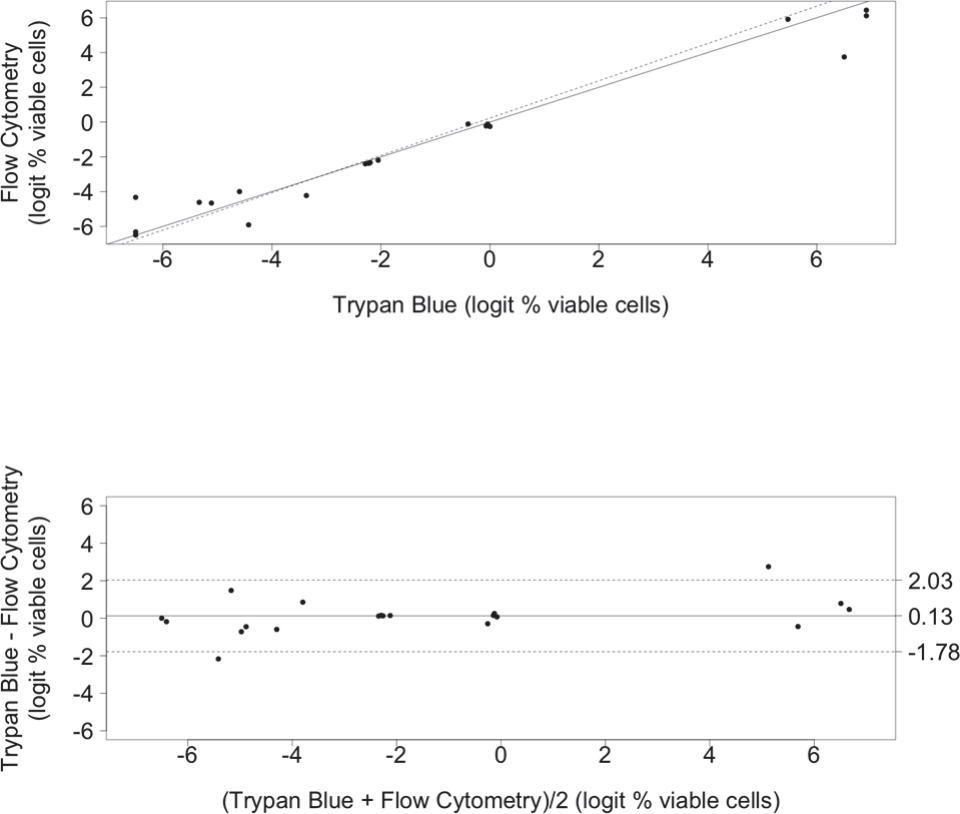
Percentage Viable Cells by Trypan Blue Staining versus Flow Cytometry. From prepared samples of 100:0, 50:50, 10:90, 1:99, 0:100 live:dead cryptococci. **4A (top). *Scatter plot for Trypan Blue and Flow Cytometry methods of measuring viable cell percentages***. Solid line indicates the reference line. Dotted line indicates the regression line. **4B (bottom). *Bland-Altman difference plot comparing the Trypan Blue and Flow Cytometry viable cell measurement methods***. The bias (mean difference between measurement methods) is shown as a solid horizontal line and the 95% limits of agreement are shown with dashed lines at +/− 1.96 standard deviations.

Using the mixtures 100:0, 50:50, 10:90, 1:99, 0:100 live:dead cryptococci at 1 McFarland–standard dilution as above, BCECF-stained non-viable cryptococci fluoresced brightly, whereas viable cells did not (Fig. 3B-F). Viable and non-viable cells were not able to be distinguished by forward scatter and side scatter alone (a typical scattergram is shown in Fig. 3A), but were distinguishable by fluorescent staining with BCECF. Since flow cytometry does not give absolute counts of live and dead cells, we compared proportions by microscopy and flow cytometry. The percentage viability correlated with the respective proportions of viable/non-viable cells, with non-viable cryptococci fluorescing more brightly than viable cells (Fig. 3B-F). For the comparison of the percentage of viable cells by cytometry versus trypan blue microscopy, data was available for n = 4 samples and 5 replicates/mixtures assessed per sample (Table 2 in S1 File). The two measures of determining the percentage of live cells were significantly linearly associated (Fig. 4A, R2 = 0.96, p<0.0001). The Bland-Altman analysis (Fig. 4B) showed a high level of agreement between the two measurement methods with a bias of-0.26 logit percentage viable cells (SD 1.68). This is consistent with the microscopy method giving a slightly higher reading than the flow cytometry method. The 95% limits of Agreement were-1.78 logit percentage viable cells and 2.03 logit percentage viable cells. Within this dataset there were two outliers.

**Figure 5:**
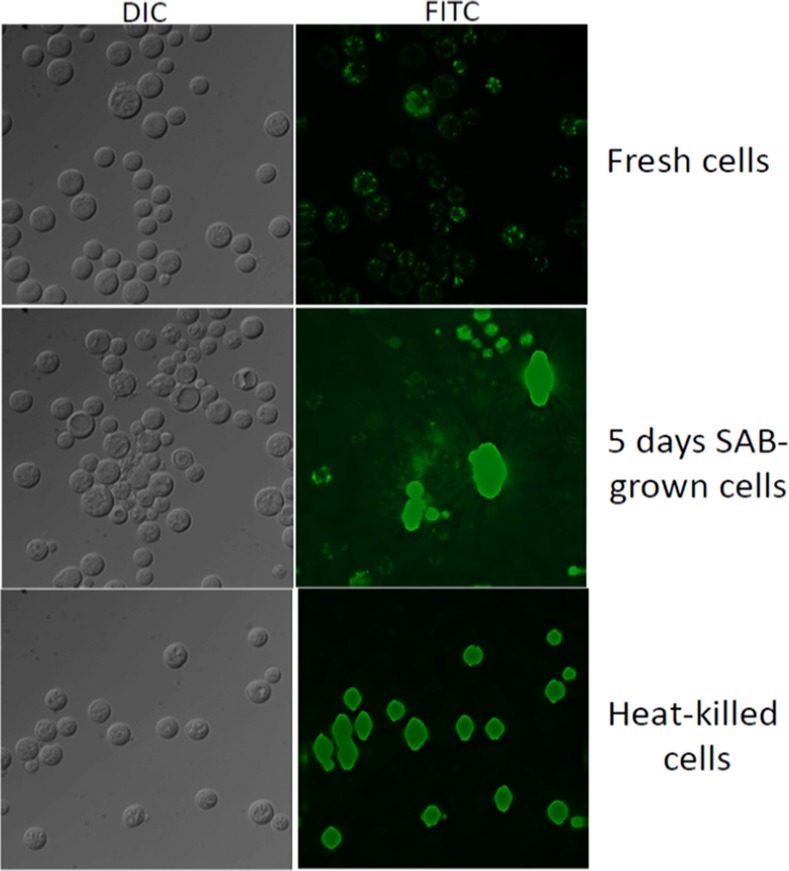
Fluorescent Microscopic Assessment of Cryptococcal Viability using BCECF. Phase contrast and fluorescence microscopic analysis of viable (fresh cells), nutrient starved cells, following 5 days growth on Sabouraud’s Dextrose Agar, and heat-killed cryptococci in the upper, middle and lower panels, respectively. DIC: Differential Interference Contrast Microscopy; FITC: Fluorescein isothiocyanate Fluorescent Microscopy. SAB: Sabouraud’s Dextrose Agar.

We noted that non-viable cryptococci fluoresced brightly whereas viable cells did not. This was contrary to the bright fluorescence in viable cells observed by others[12]. Auto-fluorescence was not responsible for this apparently reversed result since fluorescence was not observed in unstained samples. To investigate whether this result was due the method of killing, cryptococci were also killed by nutrient starvation. However, similar to heat-killed cells, starved (non-viable) cells also took up the stain to a greater extent than live (non-starved) cells as assessed by both flow cytometry (not shown) and microscopy (Fig. 5). Similar results were obtained when additional wash steps were applied to heat-killed and nutrient-starved cells post-staining. This confirmed that the preferential staining of dead cells was not due to retention of residual BCECF prior to flow analysis. Direct visualization by fluorescence microscopy (Fig. 5) confirmed that killed cells stained evenly and that viable cells stained weakly with a patchy staining pattern, consistent with compartmentalization of the dye, possibly in vacuoles.

After demonstrating good agreement between absolute cryptococcal counts by microscopy and quantitative culture and between proportions by microscopy and flow cytometry, two final experiments were also performed using flow cytometry and TruCount beads (BD Biosciences, California USA) to obtain absolute cryptococcal counts and to compare these counts to those obtained by microscopy. These demonstrated good agreement between counts obtained by microscopy and flow cytometry. These results are shown in table 1 with full data detailed in Section 3 in S1 File.

**Table 1:** Quantitation of Cryptococci by Microscopy, Quantitative Culture and Flow Cytometry.

Experiment	Microscopy count (cells/mL)	Flow cytometry^**1**^ (cells/mL)
#1^2^	3.60 × 10^6^	3.40 × 10^6^
#2^3^	8.00 × 10^6^	9.00 × 10^6^

^1^Flow cytometry counts obtained using Trucount beads.

^2^1 McFarland-standard dilution.

^3^2.85 McFarland-standard dilution.

## Discussion

This study has shown the feasibility of two rapid methods for assessment of cryptococcal viability, with the potential for implementation in a clinical laboratory setting: trypan blue exclusion microscopy and flow cytometry. Quantitative measurements of cryptococci in CSF have been correlated with CSF opening pressure, initial CSF cryptococcal antigen titres and adverse prognosis in patients with cryptococcal meningitis [4,15]. More rapid clearance of cryptococci by quantitative cultures during the first 2 weeks of therapy, measured as the EFA, has been associated with improved survival in clinical trials[1]. The resource requirement and necessary delay in obtaining results from quantitative cultures suggest that the application of rapid quantitative methods to clinical samples will provide a prognostic marker and may assist in same-day therapeutic decision-making.

Trypan blue is cheap and can be stored at room temperature. Microscopy is cheaper, quicker and less resource-intensive than quantitative cultures and does not require refrigeration or incubation of samples. It is no more difficult to perform than India ink staining and can discriminate between viable and non-viable cells. The haemocytometer is simple to use and requires only a light microscope and tubes and pipettes to mix the sample. Furthermore, in clinical CSF samples, potential confusion with leukocytes and red blood cells can be avoided by inclusion of a simple water dilution lysis step as described above. However, post-lysis, concentration of the sample by centrifugation may be required to offset the dilution effect, as it may also in samples with cryptococcal counts below a threshold of approximately 10^4^ CFU/mL.

In developed settings, trypan blue microscopy or flow cytometry offer rapid quantitation, which may assist with decisions about switch from intravenous to oral therapy and thus reduce length of stay and intravenous line-related complications. The rate of fall of viable cryptococci (the EFA) has been associated with improved survival in clinical trials [1] and work is underway to determine if this measure can be used as a marker to influence the duration of antifungal induction therapy. In developing settings, trypan blue testing may be a rapid alternative to culture in laboratories that lack culture facilities, allowing planning for hospital or outpatient management, and may be especially important in settings where patient retention and loss to follow-up are problematic.

Flow cytometry is not commonly used in clinical microbiology, but has potential applications for rapid evaluation of antimicrobial susceptibility, viability and interaction of microorganisms with human cells [12,13,25–27]. In this study, the percentage of viable cells correlated well with microscopy (Figs. 3 and 4). Additionally, use of quantitative flow cytometry (using TruCount beads) confirmed that absolute counts of cryptococci obtained by flow cytometry correlated with microscopy counts (Table 1). Previous work on killing of BCECF-AM-loaded *C*. *neoformans* strain NIH 37 by human mononuclear cells suggested that viable cryptococci retained the fluorescent form of the dye after washing[12]. Our results, which demonstrated the reverse, were not explained by auto-fluorescence, since unstained samples did not fluoresce. BCECF-AM is converted to its fluorescent form by intracellular esterases. Notably, although BCECF fluorescence is observed in viable mammalian cells, poor uptake by the yeast, *Saccharomyces cerevisiae* [25,27], and accumulation in vacuoles of *Neurospora crassa* [28], has been reported. We hypothesized that, as with *S*. *cerevisiae* [25,27], there may be inefficient hydrolysis of the AM ester by cryptococcal intracellular esterases. However, this would not explain the bright fluorescence in non-viable cells, unless esterases are activated during cell death, for example following lysosome disruption. Alternatively, the dye may be rendered fluorescent by another mechanism or uptake and retention of dye may exceed efflux in killed, compared with viable, cryptococci.

Although this was a small feasibility study using predominantly cultured samples, viability as assessed by microscopy correlated well with that obtained by quantitative culture and training in the recognition of viable and non-viable stained cells was straightforward. Similarly, quantification and viability as determined by flow cytometry agreed well with microscopy. Flow cytometry is widely available in immunology and haematology departments in resource-rich settings, but is unlikely to be available in resource-limited settings, which limits used of this technique. We did not study the techniques using very low concentrations of viable cryptococci (for which a centrifugation concentration step may be required) but did choose concentrations that are likely to be encountered in cryptococcal meningitis in clinical settings. These require further exploration in research and clinical settings before they can be recommended for clinical use.

## Conclusions

In summary, this proof-of-principle study shows the feasibility of direct microscopy using trypan blue exclusion and flow cytometry to quantify viable cryptococci in CSF samples. Trypan blue has the advantage of low cost, ready availability and stability at room temperature. The method is quick and simple to perform, requiring only a light microscope and haemocytometer, with little training. Flow cytometry also distinguished between viable and non-viable cells. These methods may be of clinical and prognostic benefit to patients and warrant validation in prospective clinical trials.

## Supporting Information

S1 File Supplemental Data. (DOCX)
