# Supplementary material for: Rapid Microscopy and Use of Vital Dyes: Potential to Determine Viability of Cryptococcus neoformans in the Clinical Laboratory
Source: PLoS One. 2015 Jan 27;10(1):e0117186. doi: 10.1371/journal.pone.0117186 (PMC4308066; doi:10.1371/journal.pone.0117186)
Supplement: S1 File — (DOCX) [file pone.0117186.s001.docx]

# Rapid microscopy and use of vital dyes to determine viability of Cryptococcus neoformans in the clinical laboratory – Supplemental Data

#### Table 1. Trypan Blue Microscopy versus Quantitative Culture

| **Sample** | **Viability (%)** | **Quantitative Culture (CFU/mL)** | **Microscopy count (CFU/mL)** |
| --- | --- | --- | --- |
| 1 | 100 | 1.38E+06 | 1.34E+06 |
| 1 | 50 | 7.80E+05 | 6.70E+05 |
| 1 | 10 | 1.40E+05 | 1.34E+05 |
| 1 | 0 | 0.00E+00 | 0.00E+00 |
| 2 | 100 | 2.90E+06 | 3.00E+06 |
| 2 | 50 | 1.40E+06 | 1.50E+06 |
| 2 | 10 | 2.47E+05 | 2.50E+05 |
| 2 | 1 | 2.51E+04 | 2.50E+04 |
| 2 | 0 | 4.80E+01 | 0.00E+00 |
| 3 | 100 | 3.50E+06 | 3.60E+06 |
| 4 | 100 | 4.22E+06 | 3.20E+06 |
| 4 | 50 | 2.11E+06 | 1.94E+06 |
| 4 | 10 | 4.83E+05 | 6.40E+05 |
| 4 | 1 | 4.89E+04 | 1.60E+05 |
| 4 | 0 | 0.00E+00 | 6.00E+01 |
| 5 | 100 | 4.11E+06 | 4.70E+06 |
| 5 | 50 | 2.51E+06 | 1.86E+06 |
| 5 | 10 | 3.94E+05 | 5.00E+05 |
| 5 | 1 | 3.95E+04 | 2.00E+04 |
| 5 | 0 | 4.38E+02 | 0.00E+00 |
| 6 | 100 | 4.02E+06 | 3.92E+06 |
| 6 | 50 | 2.04E+06 | 1.54E+06 |
| 6 | 10 | 4.07E+05 | 4.00E+05 |
| 6 | 1 | 3.64E+04 | 2.00E+04 |
| 6 | 0 | 0.00E+00 | 0.00E+00 |

#### Table 2. Percentage Viable Cells by Trypan Blue Microscopy versus Flow Cytometry

| Sample | Replicate | Microscopy (%) | Flow cytometry (%) |
| --- | --- | --- | --- |
| 1 | 1 | 100 | 97.7 |
| 1 | 2 | 50 | 43.7 |
| 1 | 3 | 10 | 8.9 |
| 1 | 4 | 1 | 1.8 |
| 1 | 5 | 0 | 1.3 |
| 2 | 1 | 99.9 | 99.84 |
| 2 | 2 | 48.26 | 44.45 |
| 2 | 3 | 11.39 | 10.02 |
| 2 | 4 | 3.33 | 1.44 |
| 2 | 5 | 1.18 | 0.27 |
| 3 | 1 | 99.58 | 99.73 |
| 3 | 2 | 48.95 | 47.18 |
| 3 | 3 | 9.23 | 8.3 |
| 3 | 4 | 0.6 | 0.94 |
| 3 | 5 | 0 | 0.15 |
| 4 | 1 | 99.9 | 99.78 |
| 4 | 2 | 40.1 | 47.28 |
| 4 | 3 | 9.8 | 8.52 |
| 4 | 4 | 0.48 | 0.98 |
| 4 | 5 | 0 | 0.18 |

#### 3. Additional Flow Cytometry: TruCount^TM^ beads

***Table 3A: Summary Table: Quantitation of Cryptococci by Flow Cytometry versus Microscopy***

| **Experiment** | **Microscopy count (cells/mL)** | **Flow cytometry^1^**  **(cells/mL)** |
| --- | --- | --- |
| #1^2^ | 3.60 x 10^6^ | 3.40 x 10^6^ |
| #2^3^ | 8.00 x 10^6^ | 9.00 x 10^6^ |

^1^Flow cytometry counts obtained using Trucount^TM^ beads.

^2^1 McFarland-standard dilution, sample also tested by quantitative culture, result 3.5x10^6^ CFU/mL (see below)

^3^2.85 McFarland-standard dilution, diluted 1/10 prior to measurement by flow cytometry.

***3B: Quantitation of Cryptococci by Flow Cytometry Experiment #1***

1 McFarland-standard dilution of live H99 *C. neoformans* in PBS harvested from culture on SDA.

Haemocytometer: 1:1 Trypan Blue added (50µL TB, 50 µL crypto suspension)

*Result =3.6x10^6^ cells/mL*

Quantitative Cultures:

100 µL crypto suspension in serial dilutions, counted day 3 (48 hours)

*Result =3.5x10^6^ CFU/mL*

Flow Cytometer:

50 µL crypto suspension added to TruCount tube and vortexed.

450 µL PBS added and vortexed. Result run in flow cytometer: (# of beads per test for lot = 49052)

Formula for calculation:

(# of events in region containing cryptococci / # of events in region containing beads) x (# of beads per test/ test volume (µL) X 1000) = crypto CFU/mL

7595 crypto events/2197 bead events x 49502 beads per test/50 µL = 3391 cells/ µL

= 3.4 x10^6^ cells/mL

*Calculated =3.4x10^6^ cells/mL*

***3C: Quantitation of Cryptococci by Flow Cytometry Experiment #2***

2.85 McFarland-standard dilution of live H99 *C. neoformans* in PBS from culture on SDA.

Haemocytometer: Initially too many to count – sample diluted one in ten.

Quantity in diluted sample=8x10^5^ cells/mL

*Calculated cryptococcal counts in original sample=8x10^6^ cells/mL*

Flow Cytometer:

50 µL cryptococcal suspension added to TruCount tube and vortexed.

450 µL PBS added and vortexed. Result run in flow cytometer: (# of beads per test for lot = 49052)

**Table 3C: *Quantitation of Cryptococci by Flow Cytometry Experiment #2***

| Sample | Dilution | Expected count | # of events in crypto region | # of events in beads region | Calculated crypto count |
| --- | --- | --- | --- | --- | --- |
| A (neat) | Undiluted | 8x10^6^ CFU/mL | error | error | Unreliable |
| B | 1/10 | 8x10^5^ CFU/mL | 4088 | 4435 | 9.0 x10^5^/mL |

*Calculated cryptococci in original sample=9x10^6^ cells/mL*
